# First cases and risk factors of super yeast *Candida auris* infection or colonization from Shenyang, China

**DOI:** 10.1038/s41426-018-0131-0

**Published:** 2018-07-11

**Authors:** Sufei Tian, Chen Rong, Hua Nian, Fushun Li, Yunzhuo Chu, Shitong Cheng, Hong Shang

**Affiliations:** Department of Laboratory Medicine, The First Affiliated Hospital of China Medical University, Shenyang, 110001 China

## Abstract

For the first time, we identified 15 cases of *Candida auris* in Shenyang, China, and then performed a risk factor assessment for these patients compared with 30 control subjects who were hospitalized in the same ward during the same period of time as the infected patients. We found that diarrhea, gastrointestinal decompression, infection, or colonization with other Candida isolates (especially *Candida albicans*) and tetracycline antibiotics were all risk factors for *C. auris* infection or colonization. Diarrhea and tetracycline antibiotics were independent risk factors. We suggest clinicians pay special attention to the emergence of multidrug-resistant *C. auris* infections or colonization.

## Introduction

We read with interest the review by Chowdhary and colleagues concerning the rapidly emerging multidrug-resistant pathogenic yeast *Candida auris*^1^, which primarily affects critically ill patients and results in significant morbidity and mortality. We noted that *C. auris* was first isolated in Japan and described as a new species in 2009^2^. In 2011, it was identified as a cause of fungemia in South Korea^3^. *C. auris* has caused serious infections globally, including in India, South Africa, Kuwait, the United Kingdom, Venezuela, the United States, Israel, Oman, and, more recently, Panama and the United Arab Emirates^4–13^. The real prevalence of *C. auris* may be underestimated because this pathogen has been routinely misidentified as *Candida haemulonii*. We therefore reviewed all isolates of Vitek-identified *C. haemulonii* obtained from a tertiary care hospital in Shenyang, China, and have found 15 misidentified cases. In the present study, we report the first cases of *C. auris* from China. Moreover, we described in detail the clinical characteristics of patients with a *C. auris* infection or colonization. Furthermore, to define the risk factors for *C. auris* infection or colonization, we conducted a matched (1:2) retrospective cohort study using patients who were neither infected nor colonized by *C. auris* as controls.

## Results

### Microbiology

**Fig. 1:**
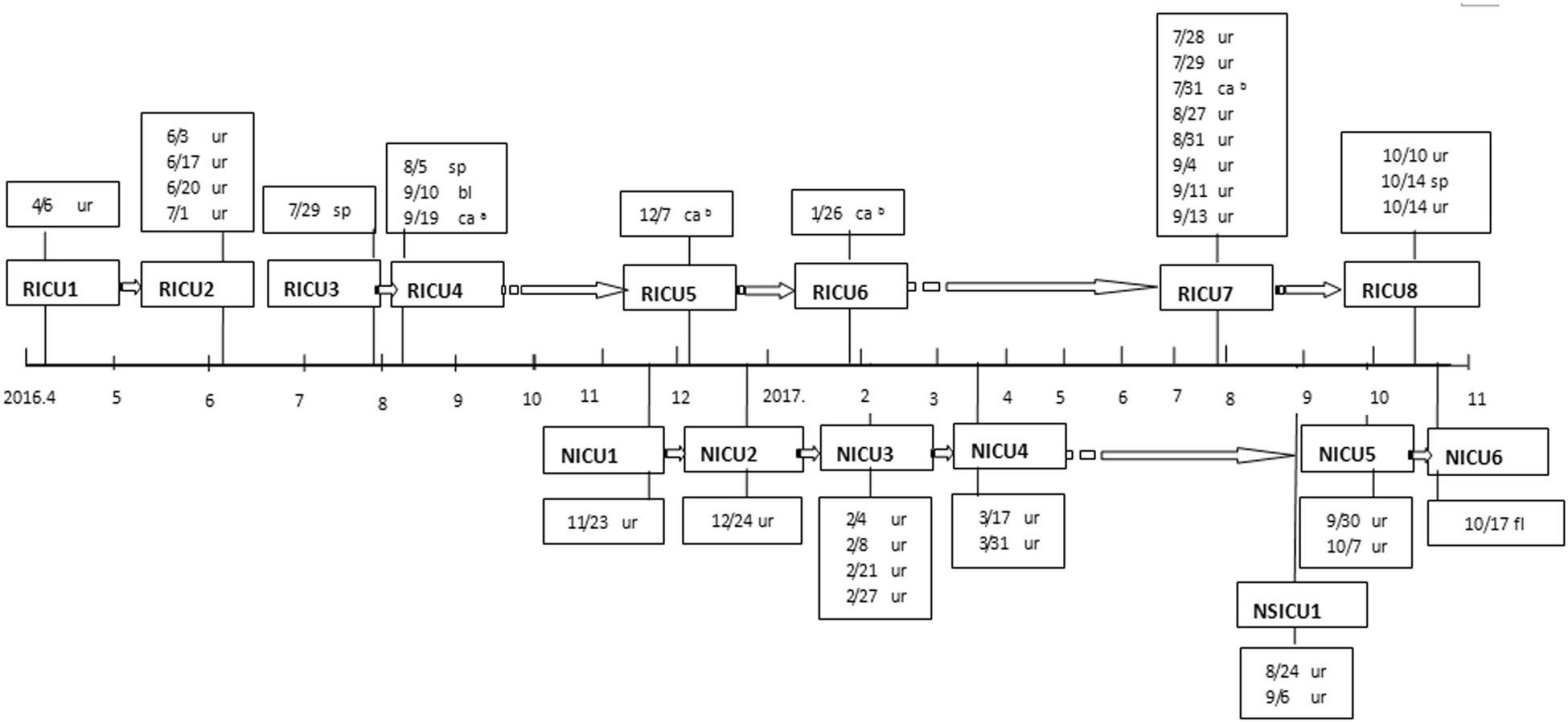
Onset chart of *Candida auris* infection. Onset chart of 15 cases of *Candida auris* infection (the detection time and sample type are shown in the textboxes); Respiratory ICU inpatient number (RICU1-RICU8); Neurology ICU inpatient number (NICU1–NICU6); Neurosurgical ICU inpatient number (NSICU1); sp sputum; ur urine; bl blood; ca^a^ central venous line; ca^b^ urinary catheter; dr drainage

During January 2011 October 2017, 35 isolates were identified as *C. haemulonii* but later confirmed as *C. auris*. A total of 26 isolates (74%) were from urine, four from a catheter, three from sputum, one from blood, and one from fluid. These isolates originated from fifteen patients (See Fig. 1). For each patient, the first isolate was selected for a follow-up study (apart from RICU4 with blood culture isolates selected).

Colonies of all isolates on Sabouraud dextrose agar were white to cream colored and smooth. They were able to grow at both 37 °C and 42 °C and had a pink colony color on CHROMagar Candida medium. We observed that organisms from all samples were oval, without pseudohyphae and germ tube formation. All samples were reidentified as *C. haemulonii* by Vitek-2 (bioMérieux, Marcy I’Etoile, France) and *Candida famata* by API 20C (bioMérieux) assays. All organisms from our isolate collections could assimilate *N*-acetyl-glucosamine in contrast to the isolates from Japan and South Korea, which was similar to Chowdhary’s observations in India reported in 2013^3^. All Vitek-identified *C. haemulonii* isolates were confirmed to be *C. auris* by the sequences of the internal transcribed spacer (ITS) region and the D1/D2 region of the large subunit (28S) of the ribosomal DNA. Then, using the genetically identified strains, an in-house super-spectrum for *C. auris* was created in the RUO (research-use-only) module in the VITEK-MS MALDI-TOF system. MALDI-TOF then could be subsequently used routinely in the laboratory for the rapid identification of *C. auris* isolates.

### Molecular characterization

All of the 15 *C. auris* isolates had 100% similarity in their ITS, D1/D2, RPB1, and RPB2 sequences. The ITS regions of our *C. auris* isolates had 100% similarity with isolates from India (GenBank accession KC692039), South Africa (GenBank accession KJ126758/ KJ126761), and Kuwait (GenBank accession LN624638) but 98% sequence homology to isolates from Japan/Korea (GenBank accession AB375772/JX459679). The D1/D2, RPB1, and RPB2 sequences of our isolates had 100% sequence homology to isolates from Japan/Korea (GenBank accession AB375773/ EU881954).

**Fig. 2:**
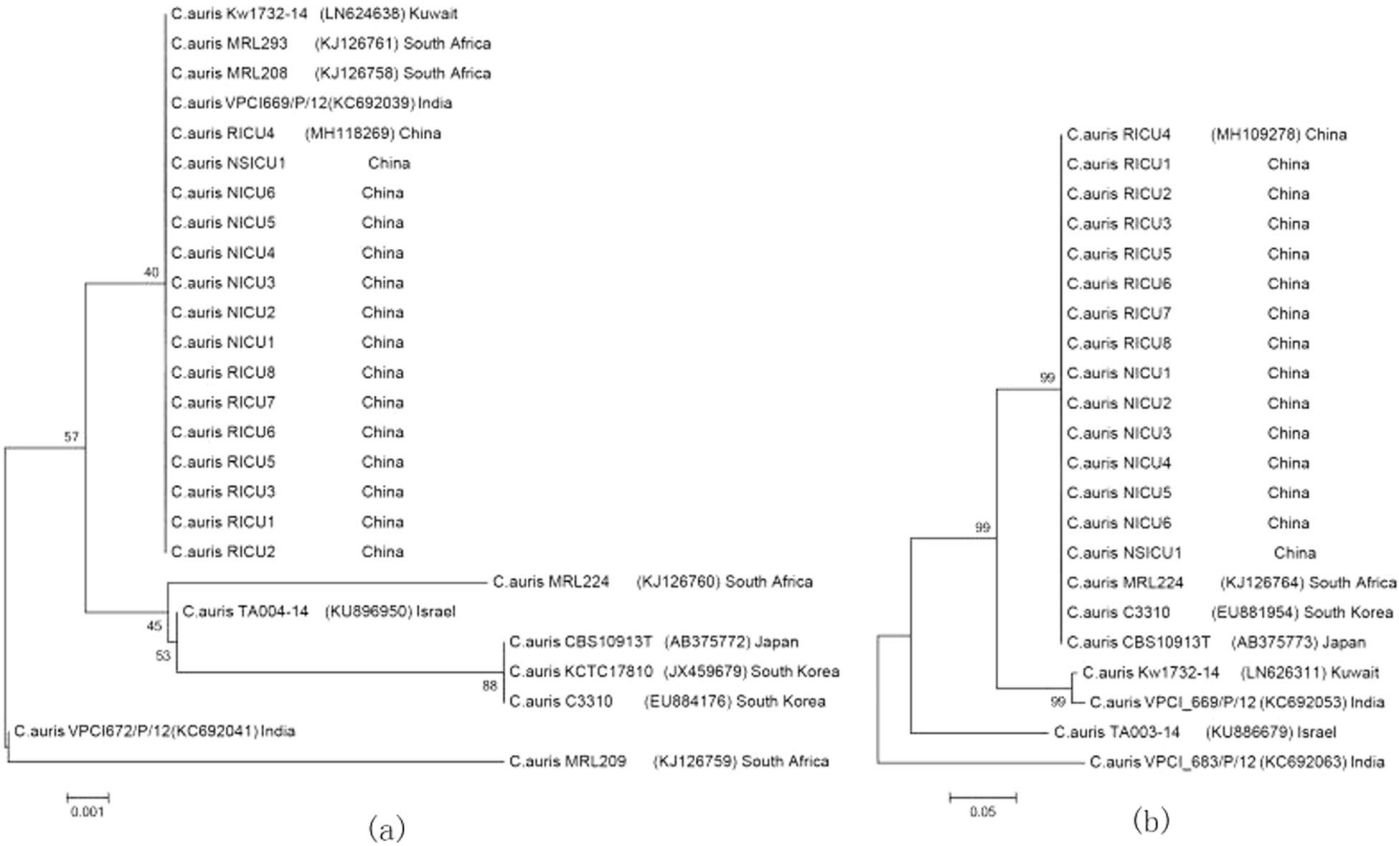
Phylogenetic relationships of *Candida auris* strains isolated in Shenyang, China, compared with reference strains. Phylogenetic trees were generated from the internal transcribed spacer (**a**) region and D1/D2 domain of the ribosomal DNA large subunit sequences (**b**). The percentage of replicate trees in which the associated taxa clustered together in the bootstrap test (1000 replicates) is shown next to each branch. Bold indicates strains from China. GenBank accession numbers are provided in parentheses. Scale bar indicates nucleotide substitutions per site

Phylogenetic trees based on the ITS and D1/D2 sequences showed that the *C. auris* isolates from China were similar to the South African clone, whereas the strain from China was distinct to some extent from other isolates from Israel, India, Japan, and South Korea (See Fig. 2).

### Antifungal susceptibility testing

All 15 isolates were resistant to fluconazole and susceptible to 5-fluorocytosine, itraconazole (IZ), voriconazole (VOR) and amphotericin B. Notably, all isolates showed high minimum inhibitory concentrations (MICs) to VOR ranging from 0.5 to 1 μg/ml. (Table 1)

**Table 1:** Information and susceptibility test of 15 isolates from 15 patients, Shenyang, China

Isolate	Age y	Sex	Source	MIC (μg/ml)								
				AMB	5-FC	FZ	IZ	VOR	PZ	CAS	MF	AND
6258	─	─	─	0.5	4	32	0.12	0.25	0.25	0.25	0.12	0.03
RICU1	70	M	Urine	0.5	<0.06	256	0.06	0.5	0.03	0.06	0.06	0.12
RICU2	69	F	Urine	0.5	<0.06	256	0.12	1	0.06	0.12	0.12	0.12
RICU3	69	F	Sputum	0.5	<0.06	256	0.06	0.5	0.03	0.06	0.06	0.12
RICU4	56	M	blood	0.5	<0.06	256	0.12	1	0.06	0.12	0.12	0.12
RICU5	82	F	Catheter	0.5	< 0.06	256	0.06	0.5	0.03	0.12	0.12	0.12
RICU6	70	M	Catheter	0.5	<0.06	128	0.12	0.5	0.03	0.12	0.12	0.12
RICU7	63	F	Urine	0.5	<0.06	256	0.12	1	0.06	0.12	0.12	0.12
RICU8	73	F	Urine	0.5	<0.06	256	0.06	0.5	0.03	0.06	0.06	0.12
NICU1	60	F	Urine	0.5	<0.06	256	0.06	0.5	0.03	0.06	0.06	0.12
NICU2	58	M	Urine	1	<0.06	256	0.12	1	0.06	0.12	0.12	0.12
NICU3	86	M	Urine	0.5	<0.06	256	0.12	0.5	0.06	0.12	0.12	0.12
NICU4	49	M	Urine	0.5	<0.06	256	0.12	1	0.06	0.12	0.12	0.12
NICU5	86	F	Urine	0.5	<0.06	256	0.06	0.5	0.03	0.06	0.06	0.12
NICU6	82	F	Drainage	0.5	<0.06	256	0.06	0.5	0.03	0.06	0.06	0.12
NSICU1	53	M	Urine	0.5	<0.06	256	0.06	0.5	0.03	0.06	0.06	0.12

*AMB* amphotericin B, *5-FC* flucytosine, *FZ* fluconazole, *IZ* itraconazole, *VOR* voriconazole, *PZ* posaconazole; *CAS* caspofungin, *MF* micafungin, *AND* anidulafungin

### The first *C. auris* case report

**Fig. 3:**
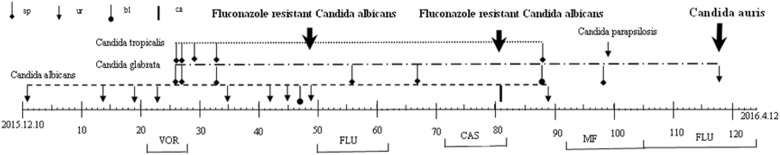
Detection time and antifungal treatment process for all candida isolates of the first patient presenting with *Candida auris* infection (RICU1) during hospitalization. This patient was treated with voriconazole (VOR 400 mg/day) for 7 days; fluconazole (FLU 400 mg/day) for 12 days; caspofungin (CAS 50 mg/day) for 10 days; and micafungin (MF 100 mg/day) for 14 days. Sp sputum; ur urine; bl blood; ca urinary catheter

In order to trace the origin of the first *C. auris* isolate in China, the complete case progress record was reported here in detail. The first case (See Fig. 3) was a 70-year-old man with a 40-year history of high paraplegia and prolonged bed rest who presented with severe pneumonia, respiratory failure, and diabetes complications. He was admitted to the Respiratory and Intensive Care Unit and then treated with empirical broad-spectrum antibiotics, a tracheotomy, an indwelling gastric tube, and urinary catheterization (the urinary catheter was changed once a month). In the hospital on day 1, a urine culture yielded *Candida albicans* (an azole-susceptible strain), which persisted on days 14, 19, 23, 35, 42, and 45. Antifungal therapy was initiated on day 21 with VOR (400 mg/day) for 7 days. Thereafter, several sputum cultures showed *Candida tropicalis* and *Candida glabrate*. On day 47, a blood culture yielded *C. albicans* (an azole-susceptible strain). On day 50, the antifungal therapy was changed to fluconazole (400 mg/day) for 12 days. During fluconazole therapy, a urine culture again yielded *C. albicans* (an azole-resistant strain) on day 49. On day 72, the antifungal treatment was changed to caspofungin (50 mg/day) for 10 days. The urinary catheter was removed on day 81, and a culture of the urinary catheter tip removed from the patient grew > 1000 colonies of *C. albicans* (an azole-resistant strain). On day 88 after admission, a blood culture yielded *C. glabrate*. On day 92, the antifungal therapy was changed to micafungin (100 mg/day) for 14 days. On day 116, the patient presented with antibiotic-associated diarrhea. On 6 April the 118th day of hospitalization and the 7th day after replacement of the urinary catheter, a urine culture yielded *C. auris* (fluconazole with MIC > 64 μg/ml). On day 124, the patient was transferred back to a local hospital for treatment.

### Clinical characteristics of *C. auris* cases

All 15 isolates were recovered from the Intensive Care Unit. Interestingly, all the cases were sporadic, but they appeared consecutively. With the exception of one case from the Neurosurgical ICU (NSICU1), 14 cases of *C. auris* infection, or colonization were inpatients in either the Respiratory and Intensive Care Unit (RICU1–RICU8) or Department of Neurology and Intensive Care Unit (NICU1–NICU6) (see Fig. 2). There was no overlap of the detection time of an infection or colonization except for the RICU7 and NSICU1 samples that came from different departments. There was a 6-month gap between the identification of the RICU6 and RICU7 cases. Cases RICU6 and RICU1 both utilized the same bed during their hospital stay (Bed No. 3), and the other two pairs of cases also used the same bed: RICU2 and RICU8 used Bed No. 7, and NICU2 and NICU6 used Bed No. 12.

All 15 patients had an indwelling urinary catheter (changed once a month), and the average duration of the indwelling urinary catheter was 35 days (range, 6~118 days). All 15 patients had an indwelling gastric tube, 14 patients utilized mechanical ventilation, 10 patients had an endotracheal tube, and 7 patients had a tracheotomy. In total, 10 of the patients had culture-positive urine at least once, and 7 patients (RICU1, RICU2, RICU7, NICU3, NICU4, NICU5, and NSICU1) developed urinary tract infections. A total of three patients (RICU8, NICU1, and NICU2) were considered possibility positive for *C. auris* colonization because of culture-positive urine samples. In addition, RICU4 was a lung cancer patient who presented with *C. auris* fungemia on the 35th day after the isolation of *C. auris* from the sputum, whereas RICU3 was also a lung cancer patient with culture-positive sputum. Moreover, NICU6 had suffered from a biliary tract infection with *C. auris*. Ten patients had immunosuppressive conditions, seven had diabetes mellitus, three had malignant tumors, and two had chronic kidney disease (Table 2).

**Table 2:** Univariate risk factor analysis of *Candida auris* infection cases vs. controls

Parameter	Cases (*n* = 15)	Controls (*n* = 30)	*P*	OR (95% CI)
Age (years)	68.0 ± 11.9	68.6 ± 15.6	0.71	
Hospital length of stay prior to infection	40.4 ± 33.4	26.9 ± 22.0	0.11	
ICU length of stay prior to infection	31.9 ± 28.5	24.1 ± 21.1	0.22	
APACHEII score on ICU admission	15.9 ± 6.2	14.9 ± 6.8	0.538	
Male	8 (53)	22 (73)	0.18	0.42 (0.11–1.52)
prognosis	6 (40)	4 (13)	0.04	4.33 (0.99–18.94)
Heart dysfunction	3 (20)	6 (20)	1.00	1.00 (0.21–4.71)
pulmonary embolism	0 (0)	3 (10)	0.21	0.90 (0.80–1.01)
pneumonia	10 (67)	23 (77)	0.48	0.61 (0.16–2.39)
Lung dysfunction	9 (60)	15 (50)	0.53	1.50 (0.43–5.27)
Malignancy	3 (20)	6 (20)	1.00	1.00 (0.21–4.71)
Diabetes mellitus	7 (47)	8 (27)	0.18	2.41 (0.66–8.81)
Chronic renal failure/hemodialysis	2 (13)	5 (17)	0.77	0.77 (0.13–4.52)
Liver dysfunction	4 (27)	2 (7)	0.06	5.09 (0.81–31.9)
Thyroid	3 (20)	0 (0)	0.01	1.25 (0.97–1.61)
Abdominal	1 (7)	0 (0)	0.15	1.07 (0.94–1.23)
Rheumatic diseases	1 (7)	1 (3)	0.61	2.07 (0.12–35.61)
central nervous system	10 (67)	19 (63)	0.83	1.16 (0.31–4.27)
Prior surgery	5 (33)	4 (13)	0.11	3.25 (0.72–14.62)
Trauma	0 (0)	2 (7)	0.31	0.93 (0.85–1.03)
Previous hospitalization	4 (27)	2 (7)	0.06	5.09 (0.81–31.9)
Abdominal drainage	3 (20)	1 (3)	0.06	7.25 (0.68–76.87)
Diarrhea	7 (47)	2 (7)	0.00	12.25 (2.11–70.99)
Mechanical ventilation	14 (93)	24 (80)	0.25	3.5 (0.38–32.14)
Central venous line	8 (53)	14 (47)	0.67	1.31 (0.38–4.52)
Arterial line	7 (47)	10 (33)	0.38	1.75 (0.49–6.21)
Urinary catheter (Foley)	15 (100)	27 (90)	0.21	0.9 (0.8–1.01)
Trachea intubation	10 (67)	17 (57)	0.52	1.53 (0.42–5.58)
Tracheostomy	7 (47)	7 (23)	0.11	2.88 (0.77–10.77)
Gastrostomy	15 (100)	28 (93)	0.31	0.93 (0.85–1.03)
Colostomy	2 (13)	2 (7)	0.46	2.15 (0.27–17.03)
Parenteral feeding	6 (40)	16 (53)	0.40	0.58 (0.17–2.05)
Bladder irrigation	0 (0)	2 (7)	0.31	0.93 (0.85–1.03)
Gastrointestinal decompression	6 (40)	1 (3)	0.00	19.33 (2.05–182.55)
Steroid treatment	5 (33)	11 (37)	0.83	0.86 (0.28–3.19)
Antineoplastic chemotherapy	1 (7)	0 (0)	0.15	1.07 (0.94–1.23)
*Candida albicans*	5 (33)	2 (7)	0.02	7.00 (1.17–42.00)
*Candida krusei*	1 (7)	0 (0)	0.15	1.07 (0.94–1.23)
*Candida tropicalis*	4 (27)	3 (10)	0.15	3.27 (0.63–17.09)
*Candida glabrate*	4 (27)	3 (10)	0.15	3.27 (0.63–17.09)
Other candida	9 (60)	7 (23)	0.02	4.93 (1.30–18.73)
Fluconazole	6 (40)	8 (27)	0.36	1.83 (0.49–6.81)
Voriconazole	2 (13)	0 (0)	0.04	1.15 (0.95–1.41)
Caspofungin	2 (13)	3 (10)	0.74	1.39 (0.21–9.33)
Micafungin	3 (20)	2 (7)	0.18	3.50 (0.52–23.70)
Antipseudomonal penicillins	7 (47)	8 (27)	0.18	2.41 (0.66–8.81)
Second-generation cephalosporins	1 (7)	5 (17)	0.35	0.36 (0.04–3.37)
Third-generation cephalosporins	12 (80)	21 (70)	0.48	1.71 (0.39–7.58)
Fluoroquinolones	7 (47)	16 (63)	0.67	0.77 (0.22–2.65)
Linezolid	3 (20)	3 (10)	0.35	2.25 (0.40–12.80)
Glycopeptides	4 (27)	5 (17)	0.43	1.82 (0.41–8.10)
Carbapenems	12 (80)	24 (80)	1.00	1.00 (0.21–4.71)
Tetracyclines	7 (47)	1 (3)	0.00	25.38 (2.71–237.6)

### Risk factor analysis

Five risk factors were statistically significant in the bivariate analysis: diarrhea, gastrointestinal decompression, the presence of other *Candida* strains, the presence of *C. albicans*, and the use of tetracycline antibiotics (i.e., minocycline or tigecycline). This study also found that diarrhea and tetracycline (minocycline or tigecycline) therapy were statistically significant in the multivariate analysis (Table 2).

## Discussion

As it caused successive epidemic outbreaks of fungemia in intensive care unit (ICU) patients in India, Europe, and the United States, multidrug-resistant *C. auris* has attracted significant concern. At present, cases of *C. auris* are being reported for the first time in Shenyang, China. Thus, we sought to analyze the risk factors that predisposed a patient for *C. auris* infection or colonization. In the present study, we analyzed 15 cases of *C. auris* infection or colonization and compared them with *C. auris* noninfected or noncolonized patients. The following factors increased the susceptibility to *C. auris* infection or colonization: diarrhea, gastrointestinal decompression, the presence of other *Candida* isolates (especially *C. albicans*), and the use of tetracycline antibiotics. Diarrhea and the use of tetracyclines were independent risk factors.

Both the univariate and multivariate analysis indicated that diarrhea was a risk factor for *C. auris* infection or colonization; gastrointestinal decompression was also a risk factor in the univariate analysis. Because the first patient experienced diarrhea before the emergence of *C. auris* infection or colonization, we inferred that the source of *C. auris* might be the gut, where colonized *Candida* species could invade via translocation and cause either localized infection or candidemia, as described by Kullberg *et al*.^14^. According to Perlin *et al*., the gastrointestinal tract is an important reservoir of resistance for Candida species, where they can form a mixed biofilm^15^. This hypothesis remains to be tested through further investigation into the intestinal microecology. *C. auris* is thought to migrate from the intestine to the urinary system, where it results in infection or colonization, as evidenced by the occurrence of Candiduria in 10 patients in this study. Another study found that *C. auris* easily aggregates in the mouse kidney, indicating that such aggregation may be a cause of an invasive infection and a continuous infection of *C. auris*. Ben-Ami *et al*. observed distinct yeast cell aggregates in the kidneys of mice with fatal *C. auris* infections, which suggests that aggregation might be a mode of immune evasion and persistence in the tissue^7^. In the present study, a urinary system infection or colonization lasted for a maximum of 47 d; therefore, it is necessary to establish a renal model of *C. auris* infection or colonization in order to pursue further research on the pathogenesis of *C. auris*.

The use of tetracycline antibiotics (tigecycline in five cases and minocycline via intranasal administration in three cases) was statistically significant in both single-factor and multi-factor analyses, but the specific mechanism remains unknown. It is worth investigating whether changes in *C. auris*’s ecological niches have brought the fungus into greater contact with susceptible humans. The occurrence of *C. auris* infections or colonization should be closely monitored during the clinical application of tetracycline antibiotics.

In addition, the first patient had infection or colonization with *C. albicans* and *C. glabrate* prior to infection or colonization with *C. auris*. According to the risk factor analysis of cases and controls, a previous *C. albicans* infection or colonization was a risk factor of *C. auris* infection or colonization. We think there are two possible reasons. (1) Infection or colonization with other *Candida* species will increase the dose of antifungal drug required for adequate treatment, and the resulting selection pressure further increases the probability of *C. auris* infection or colonization. (2) The capability of *C. auris* to form a biofilm and other virulence traits are weaker than that of *C. albicans* and other *Candida* species^16^, so maybe *C. auris* can cause infection or colonization by obtaining assistance from the invasiveness and mixed biofilm formed by other *Candida* species such as *C. albicans*. In the present study, three cases had a *C. albicans* infection or colonization before the *C. auris* infection or colonization occurred, and two cases had *C. auris* infection or colonization and *C. glabrate* infection or colonization simultaneously.

Historically, the first patient developed *C. albicans* infection with an azole-resistant strain at 49 d after admission, which lasted for 32 d. Antifungal drugs such as fluconazole, VOR, and echinocandins were administered accumulatively for 51 d, and at 118 d, *C. auris* infection was observed. Three potential causes can be identified. (1) Did horizontal drug-resistance gene transmission result in the conversion of drug-sensitive *C. albicans* to drug-resistant *C. albicans* followed by the conversion to a multidrug-resistant *C. auris* strain? We are currently pursuing studies that might answer that question. (2) The occurrence of *C. auris* infection or colonization is significantly associated with the use of antifungal drugs, and this has been confirmed by other studies. Fluconazole has been available since 1991 and echinocandins since the early 2000s; however, these drugs only recently became accessible in resource-limited settings^17^. *C. auris* was first detected in 1996, but it only became globally prevalent during the past 2 years. There is a correlation between the greater prevalence of *C. auris* and the time antifungal drug treatment was initiated. In the present study, we observed that patients with *C. auris* infection or colonization were more likely than the control group to have been treated with VOR, but no significant difference was observed because of the small sample size. Therefore, further validation with a larger sample size is required.

The first patient had undergone long-term bed rest (40 years), which does not support acquisition of *C. auris* by pathways such as foreign travel. In another study, screening for *C. auris* infection was performed on high-risk patients prior to ICU admission in *C. auris* epidemic areas. Only one subject in 2246 was found to be carrying *C. auris*^7^. Therefore, *C. auris* is not likely derived from the community but is, instead, a hospital-acquired pathogen. In the present study, the sequences of the ITS, D1/D2, RPB1, and RPB2 genes in all 15 samples were exactly identical, so we concluded that *C. auris* was likely disseminated by the same clone in our hospital. Some studies have reported that *C. auris* can survive for 28 d in the environment^18^; however, the interval of onset between the RICU6 and RICU7 samples was 6 months in the present study, suggesting that *C. auris* can survive far longer than previously reported in the environment. Further investigation is required.

Shenyang (China) is geographically adjacent to Japan and Korea, but the sequencing results for our isolates were more consistent with the isolates from South Africa. Although the reason remains unclear, we speculate that the South African strains had a higher potential for global transmission. More comprehensive studies of the Shenyang *C. auris* clone would be useful because molecular epidemiological studies may serve as a useful research model for evaluating the potential evolutionary trajectories of *C. auris* clones.

The present study has several limitations. First, the number of cases is limited, and this should be taken into consideration when interpreting the results. To date, there are only a few studies on the risk factors of *C. auris* infection or colonization. This study represents a beneficial supplement to the natural history of *C. auris* or colonization. Second, we performed a retrospective study with all the inherent problems related to that type of study design. The present study findings should be viewed only as preliminary and hypothesis-generating; they require large-scale validation. Third, subsequent research should adopt a better approach for typing methods such as amplified fragment length polymorphism or whole-genome sequencing to determine the clonality of the strains and their relationships to various global clades. Fourth, future studies that emphasize several molecular mechanisms, including resistance genes (ERG11, ERG3, FKS1, FKS2, and FKS3 genes), efflux and transporters, could provide insight about *C. auris* resistance in China. Fifth, because there was no awareness of the prevalence of *C. auris* in the present study, there were not any specific infection control measures for this pathogen. There is a requirement for attention to infection control measures to control the spread of *C. auris*.

We identified one *C. auris* sample as *C. haemulonii* by error; thus, we suspect the prevalence of *C. auris* may be underestimated via misidentification. Further epidemiological studies of *C. auris* (especially in the ICU) in China should be conducted. Clinicians and microbiologists are now facing urgent challenges stemming from the misidentification of *C. auris* and decreased susceptibility. We should all be aware of this emerging multidrug-resistant “fake *C. haemulonii*”, or *C. auris* yeast.

## Materials and Methods

### Laboratory methods

We retrospectively reviewed the microbiology records including all isolates identified as *C. haemulonii* that were later confirmed as *C. auris* during January 2011 October 2017. These organisms were reidentified by Vitek-2 (bioMérieux, Marcy I’Etoile, France), by API 20C (bioMérieux) assays and by using matrix-assisted laser desorption/ionization time-of-fight (MALDI-TOF) mass spectrometry (bioMérieux, Marcy ľEtoile, France). Subsequent molecular identification was accomplished by sequencing the ITS and D1/D2 regions. A set of four genetic loci, ITS, D1/D2, RPB1, and RPB2, were selected for a multi-locus phylogenetic analysis based on a previously published report by Cendejas-Bueno *et al.*^19^. We aligned the ITS and D1/D2 sequences of *C. auris* isolates with BioEdit and generated phylogenetic trees with the neighbor-joining method. We tested the phylogeny with the bootstrap method (1000 replicates). Evolutionary analyses were performed in MEGA6. In addition, the susceptibility of the isolates was determined using the Sensititre YeastOne colorimetric microdilution method (Thermo Fisher scientific, Oxoid, USA) in an in vitro assay according to the manufacturer’s instructions. *Candida krusei* ATCC6258 was used as the control strain. Because the breakpoints for *C. auris* were not defined, the breakpoints suggested for yeast in CLSI M27-S3 were used to interpret the MICs for this new yeast, and CLSI M27-S4 was followed for other *Candida* species^20^.

### Study design

Infected or colonized patients with Vitek-identified *C. haemulonii* that was later confirmed as *C. auris* were defined as the case group. Two control subjects hospitalized at the same time and in the same ward (ICU) were selected. These control patients had no detectable *C. haemulonii* that was later confirmed as *C. auris* in clinically significant specimens such as sputum, urine, blood, fluid, wound, or stool samples etc.

### Patient information

We retrospectively reviewed medical records of the patients including the selected cases and the control group and recorded patient demographics, hospital unit, co- morbidities, medications, and clinical characteristics. Detailed case report forms, designed specifically for this study, were prepared by the physician author (Su fei Tian) and rechecked by a second author (Chen Rong).

### Definitions

A candidaemia episode was defined by the isolation of a Candida strain from one or more blood specimen cultures drawn from a peripheral vein. For patients who had more than one episode of fungemia during the same hospitalization, only data from the first episode was analyzed. Possible *C. haemulonii* that was later confirmed as *C. auris* infection was defined as a case with a positive culture from a non-sterile site (i.e., sternal wound, urine, vascular line tip) and clinical signs and symptoms of infection requiring treatment with antifungal agents. Colonization with *C. haemulonii* that was later confirmed as *C. auris* was defined as culture-positive sputum, urine, blood, pus, wound, stool samples without clinical signs of Candida infection. Exposure to various risk factors was taken into consideration in the retrospective cohort study only if such exposure occurred prior to the development of *C. haemulonii* infection or colonization that was later confirmed as C. auris infection or colonization.

Standard criteria were used for the definition of existing co-morbidities (i.e., heart dysfunction, diabetes mellitus, chronic renal insufficiency). Steroid treatment was defined as any use of glucocorticoids (i.e., any dose for any period of time) during the hospital stay. The definition of “diarrhea” is “an intestinal disorder characterized by abnormal frequency and fluidity of fecal evacuations”^21^. According to the Bristol stool scale, Type 5 (Soft blobs with clear cut edges, passed easily), Type 6 (Fluffy pieces with ragged edges, a mushy stool), and Type 7 (Watery, no solid pieces, entirely liquid) were tending toward diarrhea. Moreover, Gram staining was used as a supportive method to help define diarrhea in this study. Gastrointestinal decompression was used to extract the gas and contents of the gastrointestinal tract through a gastric tube, thus facilitating the operation and recovery after the operation.

Exposure to various antimicrobial or antifungal agents was defined by the use of the given drugs for at least 3 consecutive days prior to the development of *C. haemulonii* infection or colonization that was later confirmed as a *C. auris* infection or colonization.

### Statistical analysis

The data are expressed as the mean ± standard deviation for continuous variables and as percentages for categorical variables. For continuous variables, Student’s *t* test or the Mann–Whitney *U* test was, respectively, used for normally and non-normally distributed variables. Categorical variables were compared by *χ*^2^ or Fischer’s exact test. Variables with *P* < 0.1 in the bivariate analysis were included in a backward stepwise multivariate logistic regression model. All statistical analyses were performed using SPSS 22.0 (SPSS Inc., Chicago, IL).

### Nucleotide sequence accession numbers

The nucleotide sequences obtained during this study were deposited in the GenBank nucleotide database under accession numbers MH118269, MH109278, MH124606, and MH124607.
